# Coccidioidomycosis as a Common Cause of Community-acquired Pneumonia

**DOI:** 10.3201/eid1206.060028

**Published:** 2006-06

**Authors:** Lisa Valdivia, David Nix, Mark Wright, Elizabeth Lindberg, Timothy Fagan, Donald Lieberman, T'Prien Stoffer, Neil M. Ampel, John N. Galgiani

**Affiliations:** * University of Arizona College of Medicine, Tucson, Arizona, USA;; † Southern Arizona Veterans Administration Health Care System, Tucson, Arizona, USA;; ‡ University of Arizona College of Pharmacy, Tucson, Arizona, USA;; § Arizona Community Physicians, Tucson, Arizona, USA

**Keywords:** Coccidioidomycosis, community-acquired pneumonia, diagnosis, research

## Abstract

The early manifestations of coccidioidomycosis (valley fever) are similar to those of other causes of community-acquired pneumonia (CAP). Without specific etiologic testing, the true frequency of valley fever may be underestimated by public health statistics. Therefore, we conducted a prospective observational study of adults with recent onset of a lower respiratory tract syndrome. Valley fever was serologically confirmed in 16 (29%) of 55 persons (95% confidence interval 16%–44%). Antimicrobial medications were used in 81% of persons with valley fever. Symptomatic differences at the time of enrollment had insufficient predictive value for valley fever to guide clinicians without specific laboratory tests. Thus, valley fever is a common cause of CAP after exposure in a disease-endemic region. If CAP develops in persons who travel or reside in *Coccidioides*-endemic regions, diagnostic evaluation should routinely include laboratory evaluation for this organism.

Coccidioidomycosis (valley fever) is an infection caused by *Coccidioides immitis* or *Coccidioides posadasii*, which are fungi endemic to parts of Arizona, California, Utah, New Mexico, Texas, Mexico, and elsewhere in Central and South America (*1*). The most common clinical syndrome resulting from infection is community-acquired pneumonia (CAP), which is characterized by systemic illness, lower respiratory tract symptoms, and various immunologic manifestations such as rashes and skeletal discomfort (*2**–**5*). This syndrome, which occurs 1–3 weeks after inhalation of a fungal spore, may be present for protracted periods. Although infection occasionally spreads hematogenously to other parts of the body, most infections eventually resolve without complications or specific antifungal therapy.

Enormous disparities exist between the predicted number of illnesses caused by coccidioidal infection and the actual number of infections reported to state departments of public health. For example, rates of skin test conversion (*6*) and the size of the susceptible population in southern Arizona indicate that illness should develop in ≈30,000 persons per year. Similar frequencies are obtained from other indirect analyses (*7*). If these estimates were accurate, coccidioidomycosis would be a common cause of CAP in this disease-endemic region. In contrast, cases reported to the Arizona Department of Health Services from 1998 to 2001 averaged <2,000 annually (*8*) or 15-fold fewer than estimated. The figure that best reflects the actual frequency of illness has profound implications for diagnostic evaluation and therapeutic management in patients with exposure to *Coccidioides* species in a disease-endemic region.

In this report, we present results of evaluations of patients seeking care for symptoms of CAP with respect to the incidence of coccidioidomycosis. Because CAP is often managed by clinicians as part of general medical practices, we chose that setting for our studies. Our findings provide the first prospective evidence that coccidioidomycosis is a common cause of CAP in the study area. In addition to the relevance of these results for residents within *Coccidioides*-endemic regions, a similar risk for coccidioidal infection should be expected for others with CAP and a recent travel history to *Coccidioides*-endemic areas.

## Methods

### Study Sample

Patients were recruited from 3 primary care sites in Tucson, Arizona: the Urgent Care Center of University Medical Center and 2 of the medical offices of Arizona Community Physicians. Recruitment was conducted during 2 time periods: from December 1, 2003, through February 21, 2004, and from May 1, 2004, through August 14, 2004. Although we sought to enroll as many eligible persons as possible during these periods, study personnel were not always available to do so.

To be eligible for enrollment, patients had to exhibit a lower respiratory syndrome of <1 month's duration that included >1 of the following: pleuritic chest pain, dyspnea on exertion, having an evaluation by a chest radiograph, multiple visits for the same respiratory problem, or administration of an antibacterial drug for presumed CAP. Patients were excluded from enrollment if they had a previously diagnosed, laboratory-confirmed coccidioidal infection, another laboratory-confirmed diagnosis for inclusion-defining illness, were <18 years of age, or had not had previous exposure >1 week in a disease-endemic area. Fewer than 5% of the patients offered enrollment refused to participate in the study.

### Study Protocol

This was an observational study, and medical management of each patient's condition remained entirely with the responsible clinician. After informed consent was obtained, persons were interviewed and their clinical records were reviewed to collect information regarding demographics, comorbid conditions, time ranges of exposure in a disease-endemic region, and recent antimicrobial therapy for current respiratory illness. Persons were asked to complete the Medical Outcomes Study 36-Item Short Form Health Survey (SF-36) (*9*), the Iowa Fatigue Scale (*10*), and a respiratory infection severity scale (*11*). Results from chest radiographs and complete blood counts, where obtained as part of routine medical care, were also recorded. A second visit was scheduled for all persons.

Serum samples were obtained at both visits. They were stored at –70°C until tested at the completion of the study. Persons identified as having a coccidioidal infection were also contacted during or within the next 6 months to determine the status of their illness.

### Serologic Analysis

Anti-coccidioidal antibodies were measured in the laboratory of 1 of the authors (J.N.G.) by several conventional methods. The double immunodiffusion technique was used to measure tube precipitin–type and complement fixing–type anti-coccidioidal antibodies (*12**,**13*). Serum samples qualitatively positive for complement fixing–type anti-coccidioidal antibodies were retested quantitatively (*14*). Anti-coccidioidal immunoglobulin M (IgM) and IgG antibodies were measured by enzyme-linked immunoassay by using a commercial kit according to the manufacturer's instructions (Coccidioides EIA-Gold, Meridian Diagnostics, Cincinnati OH, USA) (*15*). An optical density >0.20 was considered positive. The relative sensitivity of these tests in patients with coccidioidal pneumonia has been previously analyzed (*15*).

### Statistical Analysis

Patient and laboratory data were entered into a database (Access 2003, Microsoft Corp., Bellingham WA, USA), and statistical analysis was accomplished with SAS version 8.2 (SAS Institute, Inc., Cary, NC, USA). Differences between groups for categorical variables were compared with the χ^2^ test and those for continuous variables were compared with the Wilcoxon sign-rank test and Wilcoxon rank-sum test as appropriate. Differences with p values <0.05 were considered significant.

## Results

Of the 56 persons enrolled in this study, 1 did not provide a serum sample at baseline and was excluded from analysis. Fifty-five percent of the enrollees were male, and the median age was 48 years (interquartile range 33–63 years) (Table 1). Most (87%) persons were non-Hispanic whites, which reflected the demographics of the study-site sample. Patients with lower respiratory syndromes had a median of 3 additional inclusion criteria. Individual criteria were evaluation by a chest radiograph (82%), administration of an antimicrobial drug (80%), dyspnea on exertion (56%), pleurisy (44%), and multiple visits for the same condition (42%).

**Table 1:** Demographic characteristics of the study sample*

Characteristic	Total (N = 55)	Persons with valley fever (n = 16)	Others (n = 39)
Male sex, no. (%)	30 (55)	8 (50)	22 (56)
Median age, y (IQR)	48 (33–63)	47 (30–57)	48 (33–63)
Race, no. (%)			
Non-Hispanic white	48 (87)	13 (81)	35 (90)
Hispanic	4 (3.6)	2 (13)	2 (5.1)
Asian	3 (5.5)	1 (6.3)	2 (5.1)
Median body mass index (IQR)	26 (22–31)	25 (22–30)	26 (23–31)
Median length of exposure in disease-endemic area, y (IQR)†	9 (5–24)	6.5 (3.5–10)	10 (6–26)
Coexisting condition			
COPD	2	0	2
Asthma	9	1	8
Lung disease	9	2	7
History of pneumonia	4	1	3
Renal disease	2	1	1
Liver disease	1	0	1
Immunocompromised	0	0	0
Rheumatologic	1	0	1

*IQR, interquartile range; COPD, chronic obstructive pulmonary disease. †p = 0.043.

### Frequency of Coccidioidomycosis as a Cause of CAP

Of the 55 persons who provided serum samples at baseline, 19 provided second serum samples 10–40 days later (median 18 days). Of these persons, 16 were positive by >1 serologic test (29%, 95% confidence interval [CI] 16%–44%). At baseline, 12 (75%) of the 16 were positive by multiple assays, and all but 3 had positive results for multiple serologic tests for both serum samples (Table 2). Of the remaining 36 persons, all had negative results for all tests at baseline, and a second serum sample obtained from 12 persons was also nonreactive.

**Table 2:** Serologic characteristics of the study sample*

Seropositive persons (N = 16)	No.
Baseline serum sample	
Positive by >1 method	11
IDTP only	1
EIA IgM only	1
EIA IgG only	2
Negative†	1
Second serum sample	
Positive by >1 method	8
Negative	0
Not obtained	8
Seronegative persons (N = 39)	
Baseline serum sample	39
Second serum sample	12
Second serum sample not obtained	27

*IDTP, immunodiffusion tube precipitin; EIA, enzyme immunoassay; IgM, immunoglobulin M. †This person was subsequently positive by multiple assays on his second serum sample.

### Comparison of Clinical Characteristics between Groups

Demographic results for persons with and without valley fever are shown in Table 1. Length of exposure in the disease-endemic area was significantly shorter for patients with valley fever than for those who were seronegative (p = 0.043). The odds ratio for developing coccidioidomycosis in persons with exposure of <10 years to a disease-endemic area compared to those with a longer exposure time was 4.11 (95% CI 1.01–16.8). No other significant demographic differences were identified between the 2 groups. Twenty, 24, and 11 participants had age ranges of <40 years, 40–64 years, and >65 years, respectively. The percentage of participants in each of these age groups with valley fever was 30%, 29%, and 27%, respectively.

Respiratory, systemic, and musculoskeletal symptoms are shown in Table 3. Only myalgia showed a significant difference between the 2 groups. The SF-36 survey and respiratory infection severity scale did not identify additional differences. However, the Iowa Fatigue Scale survey median productivity domain score (maximum score = 10 indicates greatest reduction) was 7 for persons with valley fever compared with 4.5 for all other persons (p = 0.008).

**Table 3:** Symptoms of the study sample at enrollment

Symptoms	Persons with valley fever (n = 16), no. (%)	Others (n = 39), no. (%)	p value
Respiratory			
Cough	11 (69)	35 (90)	0.10
Sputum production	8 (50)	28 (72)	0.21
Hemoptysis	1 (6.3)	3 (7.7)	1.0
Pleurisy	9 (56)	15 (38)	0.25
Dyspnea	10 (63)	21 (54)	0.77
Systemic			
Fever	9 (56)	19 (49)	0.77
Chills	9 (56)	17 (44)	0.55
Night sweats	9 (56)	21 (54)	1.0
Fatigue	16 (100)	34 (87)	0.31
Weight loss	3 (19)	7 (18)	1.0
Musculoskeletal			
Myalgia	11 (69)	9 (23)	0.0022
Arthralgia	7 (44)	11 (28)	0.35
Rash	3 (19)	3 (7.7)	0.34

The proportion of patients in whom a chest radiograph was obtained was similar between seropositive and seronegative patients (75% vs. 72%). However, for those from whom chest radiographs were obtained (n = 40), abnormalities were significantly more frequent in participants with valley fever (75% vs. 25%, p = 0.005). Radiographic abnormalities associated with coccidioidal infection included pulmonary infiltrates in 88% and hilar adenopathy in 1 participant.

### Use of Antimicrobial Drugs

Of the 55 persons who were seen in the primary care setting, 46 (84%) were prescribed antimicrobial medications; 13 received 2 consecutive courses of treatment, and 1 received 3 consecutive courses of treatment. No differences were seen between the seropositive and seronegative groups in either the proportion treated with antimicrobial drugs (81% vs. 85%) or the proportion treated with multiple courses of drugs (31% vs. 26%).

### Follow-up of Persons with Valley Fever

All persons improved in the 6 months after enrollment. Only 1 received specific antifungal therapy (oral fluconazole for 1 month), and none required hospitalization. Eleven persons with valley fever repeated the symptom survey and the Iowa Fatigue Scale a median of 22 days after enrollment. Their responses indicated significant improvement for cough (p<0.016), fatigue (p<0.0039), cognition (p = 0.016), energy (p = 0.0015), and productivity (p = 0.062). Similar improvements were noted with the SF-36 survey and the Iowa Fatigue Scale readministered to 10 persons at the 6-month follow-up visit.

## Discussion

Of the patients enrolled in our prospective study from select ambulatory care settings within the disease-endemic region, 29% were diagnosed serologically as having coccidioidomycosis. Even if one takes into account the wide 95% CI (16%–44%), this number demonstrates a high proportion of CAP caused by this infection. Furthermore, although the serologic tests used for diagnosis are highly specific for coccidioidal infection (*12**,**13**,**16**–**18*), another study emphasized that in the first weeks of primary illness these tests frequently show negative results (*15*). In the current study, 1 of 13 participants not initially serologically positive had coccidioidal antibodies in a second serum sample. Twenty-seven persons serologically negative at enrollment did not return for retesting; thus, additional coccidioidal infections may have been identified in this group. Our results will likely provide an underestimate of the incidence in this group of patients, further strengthening the conclusion that valley fever is a common cause of CAP in persons exposed to *Coccidiodes* in a disease-endemic area.

The high frequency of valley fever as a cause of CAP found in this study is consistent with previous estimates of coccidioidomycosis as a dominant cause of CAP with exposure in disease-endemic areas. A similar estimate of 25% to 30% has been obtained retrospectively at the Southern Arizona Veterans Administration Health Care System in Tucson, Arizona (*7*). Conversely, a diagnosis of valley fever requires laboratory testing. That this practice may not be uniform among clinicians was shown in a retrospective analysis of physician-specific diagnoses at primary care clinics in Tucson, Arizona, in which the rate of diagnosing coccidioidomycosis varied between 0% and 25% among physicians within the same group practice (*7*). Similar differences might also account for the increasing case rate associated with patient age that was reported in a recent analysis of 2001 Arizona state statistics (*8*). Case rates for persons >44 years of age were nearly twice those for persons 21–44 years of age. In our study in which all persons were uniformly evaluated for valley fever, all age groups had similar rates (27.3%–30.0%). Furthermore, although not detailed in our results, severity of illness in terms of respiratory symptoms was less in elderly subjects. We interpret the differences between the state statistics and those of our study as indicating that older persons who develop an illness are more likely to have an exact diagnosis determined, underscoring underreporting of illness in some patient groups such as young adults.

A corollary to the high frequency of coccidioidomycosis seen in this study is that persons anywhere with CAP and a history of recent travel to south-central Arizona or other regions where coccidioidomycosis is highly endemic would be expected to have a similarly high risk. For this reason, obtaining a travel history for any patient with CAP is essential for early and accurate diagnosis of this disease, as well as for other regional problems such as severe acute respiratory syndrome (SARS), hantavirus pneumonia, and avian influenza. Although the Infectious Diseases Society of America practice guidelines for CAP currently recommend obtaining a complete travel history only in patients with refractory pneumonia (*19*), we recommend that the guidelines be revised to recommend obtaining a travel history at the first evaluation.

Analysis of multiple symptoms at baseline showed several characteristics associated with coccidioidal infections. Both a shorter length of exposure in a disease-endemic region and a greater frequency of radiographic abnormalities were seen in persons with valley fever compared with those without valley fever. These associations were also evident in a previous report from a university health center (*5*). Symptoms of myalgia and reduced productivity were also evident with coccidioidal infection. However, none of these associations, alone or in combination, were of a sufficient magnitude to assist clinicians in the initial diagnosis. Therefore, our findings, as in the previous study (*5*), emphasize that laboratory testing at the initial physician visit is essential to identify patients with symptoms of CAP that are caused by valley fever.

A high proportion (81%) of persons with valley fever were prescribed an initial course of antimicrobial drugs. Of these, 12 patients, 3 of whom were diagnosed with valley fever, received 2 courses of these drugs. Although diagnosis of valley fever by serologic methods is frequently delayed by 3–5 days, use of antimicrobial drugs could still be avoided or stopped earlier in patients whose illness is determined to be caused by *Coccidioides* species.

The inclusion criteria used in this study were designed both to be broadly inclusive and to select patients with more severe illness. As such, they differ in some respects from commonly used entry criteria for clinical trials of new antimicrobial drugs as treatment for CAP. For example, we chose pleuritic chest pain and dyspnea at rest and fever as an entry requirement. By using these entry criteria, we found that 3 of the 12 patients with valley fever who underwent radiographic examination had normal radiographs, which is consistent with results of a previous study (*3*), but did not adhere to Infectious Diseases Society of America or American Lung Association definitions of pneumonia (*19*). When comparing our findings to those of other studies, the way in which patients were selected should be taken into account.

Several limitations of our methods deserve emphasis. Because our inclusion criteria were not standard, comparison of our results to those of other studies of CAP is difficult. Also, we did not have a diagnosis for patients without valley fever. Since this is a relatively small study, additional expanded studies may be useful, especially to extend observations to other groups such as children, the elderly, those requiring hospitalization, and residents elsewhere within disease-endemic regions. Future studies are also needed to determine best practices for management of primary coccidioidal infection and possible therapy with specific antifungal treatment. Such a high proportion of CAP caused by *Coccidioides* species should provide further impetus to conduct those studies.
