# Correction: Vol. 12, No. 6

**DOI:** 10.3201/eid1208.C21208

**Published:** 2006-08

**Keywords:** Correction Vol. 12, No. 6, Valdivia

In "Coccidioidomycosis as a Common Cause of Community-acquired Pneumonia," by Lisa Valdivia et al., an error occurred in the next-to-last paragraph of the Discussion section. The fourth sentence of the paragraph should read "By using these entry criteria, we found that 3 of the 12 patients with valley fever who underwent radiographic examination had normal radiographs, which is consistent with results of a previous study (*3*), but did not adhere to Infectious Diseases Society of America or American Lung Association definitions of pneumonia (*19*)."

The corrected text appears in the online article at http://wwwnc.cdc.gov/eid/article/12/6/c2-1208_article.htm.

We regret any confusion these errors may have caused.

